# GBS-SNP-CROP: a reference-optional pipeline for SNP discovery and plant germplasm characterization using variable length, paired-end genotyping-by-sequencing data

**DOI:** 10.1186/s12859-016-0879-y

**Published:** 2016-01-12

**Authors:** Arthur T. O. Melo, Radhika Bartaula, Iago Hale

**Affiliations:** College of Life Sciences and Agriculture, Department of Biological Sciences, University of New Hampshire, Durham, NH USA; College of Life Sciences and Agriculture, Genetics Graduate Program, University of New Hampshire, Durham, NH USA

**Keywords:** GBS, GBS-SNP-CROP, SNP genotyping, Orphan crops, Plant genetic resources, Core collections

## Abstract

**Background:**

With its simple library preparation and robust approach to genome reduction, genotyping-by-sequencing (GBS) is a flexible and cost-effective strategy for SNP discovery and genotyping, provided an appropriate reference genome is available. For resource-limited curation, research, and breeding programs of underutilized plant genetic resources, however, even low-depth references may not be within reach, despite declining sequencing costs. Such programs would find value in an open-source bioinformatics pipeline that can maximize GBS data usage and perform high-density SNP genotyping in the absence of a reference.

**Results:**

The GBS SNP-Calling Reference Optional Pipeline (GBS-SNP-CROP) developed and presented here adopts a clustering strategy to build a population-tailored “Mock Reference” from the same GBS data used for downstream SNP calling and genotyping. Designed for libraries of paired-end (PE) reads, GBS-SNP-CROP maximizes data usage by eliminating unnecessary data culling due to imposed read-length uniformity requirements. Using 150 bp PE reads from a GBS library of 48 accessions of tetraploid kiwiberry (*Actinidia arguta*), GBS-SNP-CROP yielded on average three times as many SNPs as TASSEL-GBS analyses (32 and 64 bp tag lengths) and over 18 times as many as TASSEL-UNEAK, with fewer genotyping errors in all cases, as evidenced by comparing the genotypic characterizations of biological replicates. Using the published reference genome of a related diploid species (*A. chinensis*), the reference-based version of GBS-SNP-CROP behaved similarly to TASSEL-GBS in terms of the number of SNPs called but had an improved read depth distribution and fewer genotyping errors. Our results also indicate that the sets of SNPs detected by the different pipelines above are largely orthogonal to one another; thus GBS-SNP-CROP may be used to augment the results of alternative analyses, whether or not a reference is available.

**Conclusions:**

By achieving high-density SNP genotyping in populations for which no reference genome is available, GBS-SNP-CROP is worth consideration by curators, researchers, and breeders of under-researched plant genetic resources. In cases where a reference is available, especially if from a related species or when the target population is particularly diverse, GBS-SNP-CROP may complement other reference-based pipelines by extracting more information per sequencing dollar spent. The current version of GBS-SNP-CROP is available at https://github.com/halelab/GBS-SNP-CROP.git

**Electronic supplementary material:**

The online version of this article (doi:10.1186/s12859-016-0879-y) contains supplementary material, which is available to authorized users.

## Background

The conservation and utilization of plant genetic diversity is regularly cited as a critical strategy in meeting the growing global food demand [1]. For the handful of truly global crops that provide the vast majority of the world’s caloric and protein intake (e.g. wheat, rice, maize, soybean, palm) [2], extensive resources exist to facilitate such ongoing improvement, including well-characterized gene/seed banks, international communities of researchers, and vast collections of genetic and genomic resources. Rightly, the call for ongoing investment in such resources continues [3]. For more minor agricultural plant species, however, particularly those of unique or limited relevance to developing countries, relatively fewer resources exist, leading to the designation of such species as underutilized, neglected, or orphan crops [4]. In West Africa alone, examples of such species abound and include cereal grains (e.g. *Digitaria exilis*), leafy vegetables and seed crops (e.g. *Telfairia occidentalis*), legumes (e.g. *Sphenostylis stenocarpa*), tuber crops (e.g. *Plectranthus rotundifolius*), corm crops (e.g. *Colocasia esculenta*), fruit trees (e.g. *Annona senegalensis*), oil nut trees (e.g. *Vitellaria paradoxa*), and herbs (e.g. *Hibiscus sabdariffa*). Though historically under-researched, orphan crops are now recognized as germane to the issue of future global food security due to their potential to diversify the food supply [5], enhance the micronutrient content of people’s daily diets [6], perform favorably under local and often extreme environmental conditions [7], and improve the overall environmental sustainability of smallholder agricultural systems [8].

Increasingly rapid and inexpensive genome-wide genotyping methods, enabled by ever improving next generation sequencing (NGS) platforms, have revolutionized trait development, breeding, and germplasm curation in the global crops [9]; and the potential for such genome-enabled improvement of orphan crops is clear. By virtue of its simple library preparation and robust approach to genome reduction, genotyping-by-sequencing (GBS) [10] in particular has emerged as a cost-effective strategy for genome-wide SNP discovery and population genotyping. The objective of GBS is not merely to discover SNPs for use in a fixed downstream assay (e.g. SNP-chip) but rather to simultaneously discover such polymorphisms and use them to genotype a population of interest. By combining the power of multiplexed NGS with enzyme-based genome complexity reduction, GBS is able to genotype large populations of individuals for many thousands of SNPs for well under $0.01 per datapoint [11, 12]. Shown to be robust and flexible across a range of species and populations, GBS has become an important tool for genomic studies in plants, yielding molecular markers for genetic mapping [12], genomic selection [13], genetic diversity studies [14, 15], germplasm characterization [16–18], cultivar identification [19–21], and conservation biology and evolutionary ecology studies [22].

To date, relatively little effort has been devoted to developing high-performing GBS pipelines in the absence of a reference genome [23], perhaps in part due to the assumption that a low-quality reference of any plant species is now affordable enough to be within the reach of interested programs [24, 25]. For severely under-resourced curation, research, and breeding programs for orphan crops, however, such an assumption may not hold. Although great effort is underway to muster the resources necessary to develop foundational genomics resources like annotated reference genomes for some orphan crop species (e.g. the African Orphan Crops Consortium) [26], such efforts are necessarily targeted and narrow in scope relative to the estimated 80,000 edible plant species around the world, of varying relevance to local diets [27–29]. For many orphan crop species, therefore, a reference-free GBS pipeline could be of great value, enabling access to the per-genotype cost-effectiveness of GBS without the up-front and often prohibitive cost of a reference genome.

Here, we describe an efficient pipeline for SNP discovery and genotyping using paired-end (PE) GBS data of arbitrary read lengths to facilitate genetic characterization, whether or not a reference genome is available. Executed via a sequence of Perl scripts, this GBS SNP-Calling Reference Optional Pipeline (GBS-SNP-CROP) integrates custom parsing and filtering procedures with well-known, vetted bioinformatic tools, giving users full access to all intermediate files.

## Results and discussion

In this section, we explain the GBS-SNP-CROP workflow in detail and discuss its strategies for maximizing data usage and distinguishing high-confidence SNPs from both sequencing and PCR errors. Finally, we present data on its favorable performance relative to the reference-based TASSEL-GBS [30] and network-based (i.e. reference-independent) TASSEL-UNEAK [15] pipelines for a sample dataset consisting of 150 bp PE GBS reads for a library of 48 diverse accessions of cold-hardy kiwiberry (*Actinidia arguta*), an underutilized tetraploid horticultural species.

### The GBS-SNP-CROP workflow

**Fig. 1:**
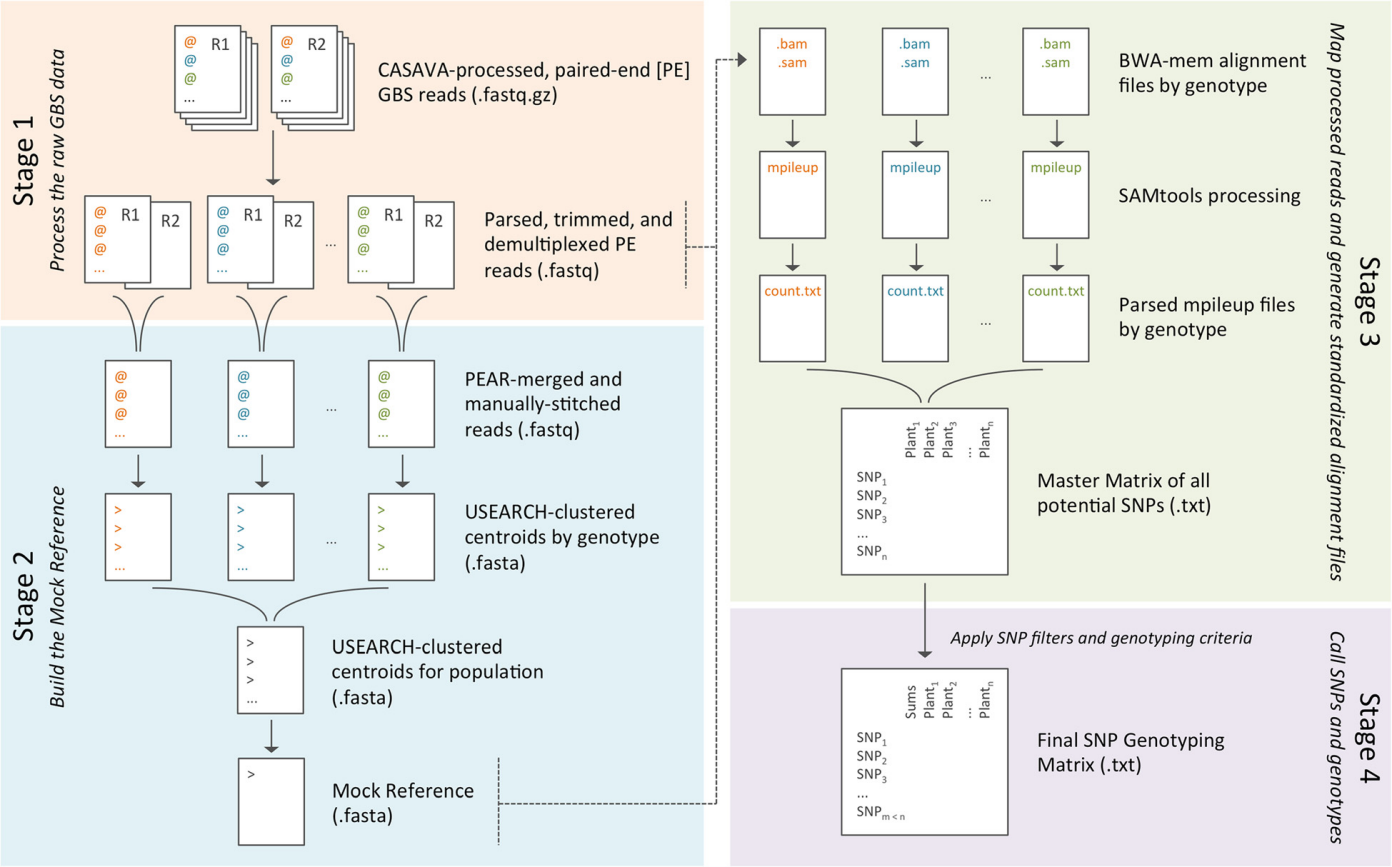
Schematic of the four stages of the SNP-GBS-CROP workflow

The GBS-SNP-CROP workflow can be divided conceptually into four main stages: (1) Process the raw GBS data; (2) Build the Mock Reference, if a reference genome is unavailable; (3) Map the processed reads and generate standardized alignment files; and (4) Call SNPs and genotypes (Table 1; Fig. 1). In this section, we explain how these stages are accomplished within GBS-SNP-CROP, with particular emphasis on the rationale throughout. While the relevant Perl scripts are referenced in this discussion, please refer to the GBS-SNP-CROP User Manual for the details of pipeline execution (https://github.com/halelab/GBS-SNP-CROP.git).

**Table 1:** Outline of the GBS-SNP-CROP workflow, featuring inputs and outputs of all seven steps (scripts)

	Input file(s)	Output file(s)	Time^a^ (hrs:mins)
Stage 1. Process the raw GBS data			
*Step 1* Parse the raw reads	- CASAVA generated paired-end (R1, R2) files (.fastq.gz)	- Parsing summary information (.txt)	2:24
		- Read length distribution summary (.txt)	
	- Barcode-ID file (.txt)	- Parsed paired-end [PE] reads (.fastq)	
		- Parsed, unpaired R1 reads (.fastq)	
*Step 2* Trim based on quality	- Parsed PE reads (.fastq)	- High quality, parsed PE reads (.fastq)	0:10
		- High quality, parsed singletons (.fastq)	
*Step 3* Demultiplex	- One pair (R1, R2) of high quality files (.fastq) per library	- One pair (R1, R2) of high quality files (.fastq) per genotype	0:16
	- Barcode-ID file (.txt)		
Stage 2. Build the Mock Reference			
*Step 4* Cluster reads and assemble the Mock Reference [MR]	- Genotype-specific PE files (.fastq)	- Mock Reference [centroids] (.fasta)	0:14^b^
	- Barcode-ID file (.txt)	- Mock Reference [genome] (.fasta)	
Stage 3. Map the processed reads and generate standardized alignment files			
*Step 5* Align with BWA-mem and process with SAM tools	- Genotype-specific high quality PE files (.fastq)	- Filtered reads (.bam)	3:36
		- Sorted BAM files (.sorted.bam)	
	- Reference or MR [genome] (.fasta)	- Indexed BAM files (.sorted.bam.bai)	
	- Barcode-ID file (.txt)	- Indexed reference or MR (.fasta.idx)	
		- One base call alignment summary file (.mpileup) per genotype	
*Step 6* Parse mpileup output and produce the SNP discovery master matrix	- One base call alignment summary file (.mpileup) per genotype	- One base call alignment summary count file (.txt) per genotype	4:37
	- Barcode-ID file (.txt)	- SNP discovery master matrix (.txt)	
Stage 4. Call SNPs and Genotypes			
*Step 7* SNP genotyping across the population	- SNP discovery master matrix (.txt)	- SNP genotyping matrix for the population (.txt)	0:04

^a^ The computation times presented here are specific to the particular dataset in this study

^b^ The time to build the Mock Reference using only the single most read-abundant genotype (-MR01). Using the five most read abundant genotypes and using all 48 genotypes, the required computation time for this step increases to 0:55 and 4:30, respectively (see Table 2)

#### Stage 1. Process the raw GBS data

As written, the code associated with Step 1 (“Parse the raw reads”; see Table 1) is compatible with Illumina1.8+ sequencing data, where the input files are assumed to be CASAVA-processed, paired-end (i.e. R1 and R2), and compressed FASTQ files (*.fastq.gz). As per the protocol developed by Poland et al. [11], these FASTQ files are assumed to contain multiplexed reads from a barcoded library of genotypes, where the R1 read begins with a 6–10 bp barcode followed by the restriction site of the less-frequent cutter (e.g. *PstI*); and the R2 read begins with the restriction site of the more-frequent cutter (e.g. *MspI*). To execute this stage of the pipeline, an auxiliary text file is required that associates each barcode with its corresponding genotype ID (see example “Barcode-ID” file in Appendix A of the GBS-SNP-CROP User Manual).

The script for Step 1 processes the raw reads in a relatively standard manner, beginning by searching the R1 read for a high-confidence barcode sequence (i.e. no more than one mismatch, relative to the provided list of barcodes) immediately preceding the expected cut site remnant of the less frequent cutter. If both barcode and cut site are found, they are trimmed from the read, the barcode is appended to the headers of both the R1 and R2 reads, and the pair is retained for further processing. This first parsing script then searches for the 3′-ends of each GBS fragment, indicated by the in-line presence of the Illumina common adapter coupled with the appropriate cut site residue. If found, the reads are truncated appropriately. Finally, all reads consisting of a majority of uncalled bases (i.e. N’s) are discarded.

Further read trimming based on user-specified minimums for both Phred quality score and read length is done in Step 2, using the bioinformatics tool Trimmomatic [31]. Finally, in Step 3, all parsed and quality-filtered reads are processed according to their barcodes; and genotype-specific FASTQ files are produced for all genotypes. The final output of Stage 1 is a pair (R1 and R2) of FASTQ files for each genotype, containing all parsed and quality-filtered reads for downstream analysis.

#### Stage 2. Build the Mock Reference

If a suitable reference genome is available for the target population, one may move directly to Stage 3 of the pipeline. If such a reference is unavailable, however, the parsed and quality-filtered reads from Stage 1 are used to build a GBS-specific, reduced-representation reference (hereafter “Mock Reference”) to enable GBS read mapping and facilitate SNP discovery. This stage of the pipeline relies upon a similarity-based clustering strategy to group the GBS reads, first within- and subsequently (if desired) across-genotypes, in order to generate representative reference sequences for the full set of GBS fragments.

To begin, the pipeline calls upon the PEAR software package [32] to merge the processed paired-end reads into single reads spanning the complete GBS fragment lengths, wherever sequence overlap for a pair is sufficient (≥10 bp) to justify merging. For each genotype selected to contribute to the Mock Reference (see “GBS-SNP-CROP Performance”), this step generates three different FASTQ files: An “assembled” file, containing successfully merged reads, and two “unassembled” files (R1 and R2), comprised of sequentially-paired R1 and R2 reads that could not be merged, due in part to a lack of sufficient overlap because of long GBS fragment lengths. Next, the pipeline stitches together all unmerged reads by joining pairs of sufficiently long “unassembled” R1 and R2 sequences together with an intermediate run of 20 high-quality A’s, thus producing a FASTQ file of “stitched” R1 + R2 reads. Representing the reduced genomic space targeted by the GBS restriction protocol, these PEAR-assembled and manually-stitched reads are then concatenated into a single FASTQ file per genotype for use in building the Mock Reference.

Next, GBS-SNP-CROP calls upon the USEARCH software package [33] to cluster these “assembled” and “stitched” reads based on a user-specified similarity threshold, thereby producing a reduced list of non-redundant consensus sequences (centroids) that span the GBS fragment space. To accomplish this, the USEARCH clustering procedure is executed first *within* each selected genotype (i.e. USEARCH clusters “assembled” and “stitched” reads into sets of genotype-specific centroids) and subsequently, if more than one genotype is selected to build the Mock Reference, across all selected genotypes (i.e. USEARCH clusters all genotype-specific centroids into a master set of centroids for the population). Representing the sampled GBS data space for the population, it is this resultant set of non-redundant consensus sequences that comprises the Mock Reference genome for subsequent mapping. Depending on the intended use of the resultant genotypic data (e.g. diversity characterization, linkage map construction, trait association, etc.), the similarity threshold specified for USEARCH may be adjusted to collapse homologous regions or maximize their discrimination, an issue of particular relevance in polyploid species.

In the end, Stage 2 produces two different Mock Reference FASTA files. The first (“MockRef_Genome.fasta”) consists of a single, long FASTA read comprised of all the centroids identified above, linked together into one contiguous sequence. The second (“MockRef_Clusters.fasta”) contains the same centroids in the same order, but in this case the centroid boundaries are preserved because each centroid exists as a separate FASTA entry. While the former file is used as the Mock Reference for read alignment (see next section), the latter is useful for optional downstream SNP filtering and analysis.

#### Stage 3. Map the processed reads and generate standardized alignment files

To align the processed reads from Stage 1 to the reference, whether a true reference genome or a Mock Reference built in Stage 2, GBS-SNP-CROP again relies upon familiar bioinformatics tools, in this case BWA [34] for alignment and SAMtools [35] for manipulating and processing the alignment output. Specifically, the BWA-mem algorithm is used to align the processed reads, genotype-by-genotype, to the reference. SAMtools is then called upon to accomplish the following steps: 1) Filter the mapped reads via SAMtools flags, retaining only those which map appropriately as pairs without potentially confounding secondary or supplementary alignments (see the GBS-SNP-CROP User Manual for more detail); 2) Convert the filtered SAM files to BAM files; 3) Index and sort the BAM files; 4) Index the FASTA reference sequence; and 5) Produce a base call alignment summary (mpileup file) for each genotype. These six steps (BWA-mem alignment and the five SAMtools procedures) are carried out individually for each genotype, with the Step 5 script automating the process.

In Step 6, the genotype-specific mpileup files are distilled into “count” text files containing four essential tab-delimited columns: (1) Reference genome/chromosome identifier; (2) Base position; (3) Reference base at that position; and (4) A comma-delimited string containing aggregated alignment information at that position (i.e. depths of A, C, G, and T reads). Each count file is then parsed, with only those rows containing reads polymorphic to the reference sequence kept, thereby generating liberal genotype-specific lists of potential SNP positions, with full read depth information retained. It is during this mpileup parsing that all putative indels are rigorously detected and excluded from downstream variant calling, thus making GBS-SNP-CROP a SNP-exclusive pipeline.

Once the mpileup parsing is completed for each genotype separately, Step 6 proceeds by mining the full set of resultant genotype-specific count files to generate a single, non-redundant master list of all potential SNP positions throughout the target population. Alignment information is then extracted from the original count files for each genotype for all potential SNP positions in the master list and the data organized into a SNP discovery “master matrix” for the entire population. By capturing both genotype-specific (columns) and population-level (rows) alignment data in one table, the master matrix is a powerful and streamlined summary of the GBS data that contains the essential information to not only distinguish high-confidence SNPs from likely sequencing and PCR errors but also to make subsequent genotype calls using stringent depth criteria, as explained in the next section.

#### Stage 4. Call SNPs and genotypes

Once generated, the master matrix is systematically pared down via a series of SNP-culling filters to arrive at a final “SNP genotyping matrix” containing only high-confidence SNPs and genotypes. To begin, the master list of potential SNPs is parsed based upon a flat criteria of independence, namely that a SNP is retained for further consideration if and only if there exist independent instances of the putative secondary allele, at a specified minimum depth (e.g. 3), across at least three genotypes. This simple requirement for independent occurrences of the less-frequent allele is an essential strategy for minimizing false SNP declarations due to random sequencing and PCR errors, including strand bias errors [36].

Next, GBS-SNP-CROP advances only potential bi-allelic SNPs (i.e. it excludes multi-allelic variants) by imposing a population-level allele frequency filter via a user-defined Alternative Allele Strength parameter (-altStrength, Step 7). For each potential SNP position, this parameter considers the total read depth, across the whole population, of all four bases, from primary (the allele with the highest depth at that position) to quaternary (the allele with the lowest depth). A potential SNP is retained for further downstream analysis if and only if it is strongly bi-allelic, that is if:

$$ \frac{2{}^{\circ}\  Allele\  Depth}{2{}^{\circ}\  Depth+3{}^{\circ}\  Depth + 4{}^{\circ}\  Depth}> altStrength $$

For a tetraploid species, we suggest a minimum value of 0.90 for this parameter, though higher values may be imposed in the interest of stricter error control (see Additional file 1).

After these initial basic population-level culling procedures, genotypic states (primary homozygote, heterozygote, or secondary homozygote) are assigned for all remaining SNP-accession combinations. To call a heterozygote, a given genotype must have a user-specified minimum read depth for each allele (e.g. 3); and the read depth ratio of the lower-coverage to higher-coverage allele must exceed a user-specified, ploidy-appropriate threshold (e.g. 0.1; see Additional file 1). If the ratio falls below this minimum threshold, GBS-SNP-CROP refrains from making a genotypic assignment (i.e. the genotype is designated as missing data). The GBS-SNP-CROP genotyping criterion for homozygotes is more stringent, requiring a relatively high, user-specified minimum depth (e.g. ≥11 when the secondary allele count is zero and ≥48 when the secondary allele count is one; see Additional file 1) in an effort to reduce the rate of erroneous calls (i.e. true heterozygotes called as homozygous due to sampling bias). Finally, in an effort to retain only broadly informative SNPs, the matrix is further reduced such that all SNPs (i.e. rows) are discarded for which more than some user-specified maximum of genotypes are without genotypic calls, either because read depth = 0 or genotypic states were unassignable due to the criteria discussed above.

**Fig. 2:**
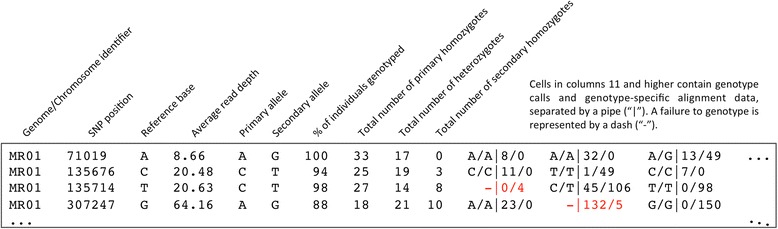
Structure of the final SNP genotyping matrix. As shown here, the GBS-SNP-CROP final genotyping matrix contains summary statistics as well as complete genotype-specific alignment data for each SNP called. The cells in red represent instances in which a genotypic state could not be assigned, either due to insufficient read depth (-|0/4) or a read depth ratio outside of the user-specified acceptable range (-|132/5)

To facilitate the downstream characterization of the high-confidence SNPs that pass all the above filters, the final SNP genotyping matrix contains both summary statistics as well as complete genotype-specific alignment data for each retained SNP. As shown in Fig. 2, the first ten columns of the matrix feature the following information: 1) Genome/chromosome identifier; 2) SNP position; 3) Reference base; 4) Average read depth at that SNP position across the population; 5) Primary allele (i.e. the most frequent allele at that position, based on read depth across the population); 6) Secondary allele (i.e. the less frequent, or alternative, allele at that position); 7) Percentage of individuals from the population genotyped for that SNP; 8) Total number of homozygotes for the primary allele; 9) Total number of heterozygotes; and 10) Total number of homozygotes for the secondary allele. Columns 11 and higher contain the complete alignment data for each individual genotype for all possible SNP positions. The ability of GBS-SNP-CROP to consider both genotype-specific and population-level alignment data simultaneously through the master matrix during the processes of SNP filtering and genotyping is an essential feature of the pipeline and motivates its disuse of Minor Allele Frequency (MAF), a problematic filtering parameter when attempting to characterize broadly diverse germplasm collections, as opposed to more closely-related breeding populations.

#### Other downstream tools

In addition to the scripts associated with the core GBS-SNP-CROP workflow described above, one additional script (“GBS-SNP-CROP-8.pl”) is provided to facilitate downstream management of the final SNP genotyping matrix by enabling users to convert the matrix into formats compatible with the familiar statistical analysis software packages R [37], Tassel GUI [30], and PLINK [38]. Specifically, the script produces a genotype matrix appropriate for diversity analyses within R (e.g. calculating distance metrics, generating cladograms, etc.) by replacing primary homozygotes with 0, heterozygotes with 0.5, secondary homozygotes with 1, and unassigned genotypes with “NA”. It can also transform the final SNP genotyping matrix into a HapMap file for use as input into Tassel GUI, allowing users to easily access the functionality of that software package for forward analysis, or create the transposed . PED file required by the whole genome association analysis toolset PLINK.

### Avoiding false SNP calls

One well-recognized challenge posed by NGS data is the rate of erroneous base calls produced, rates which vary across both platforms and base position within reads. For instance, the error rate of current Illumina sequencing platforms ranges from 1 to 10 bases per kilobase sequenced, with errors concentrated in the beginnings and ends of reads (i.e. tail distance bias) [39, 40]. With typical sequencing runs producing billions of base calls (e.g. a single HiSeq 2500 Illumina flow cell can produce as much as 400 Gb of data [41]), there is real potential for millions of errors that can confound analysis [42]. Del Fabbro et al. [43] discuss the importance of quality trimming to increase the reliability of downstream analysis, with simultaneous gains in terms of both computational resources and time. While other authors assert that quality scores may not be perfectly reliable indicators of true nucleotide quality [44, 45], GBS-SNP-CROP begins with a stringent recognition of barcodes (Hamming distance ≤1) and cut sites (no mismatches), followed by trimming based on Phred score.

In addition to this basic quality filtering of the raw reads, the pipeline seeks to minimize false SNP calls through its approach to SNP discovery and filtering. First, only those reads that map as paired-ends without secondary or supplementary alignments to the reference are retained. Additional parameters are called upon within the SAMtools mpileup algorithm to avoid false SNPs due to misalignment and excessive mismatches (see the GBS-SNP-CROP User Manual). SNPs that pass the above filters must then also satisfy the aforementioned requirement of independence, assessable by virtue of the unique format of the GBS-SNP-CROP master matrix. By leveraging both genotype-specific and population-level depth information, this requirement effectively reduces the probability of calling false SNPs due to both sequencing and PCR errors, including strand bias errors, since the exact same errors must arise independently, at depth, across multiple genotypes. GBS-SNP-CROP also makes use of stringent genotyping criteria to further reduce the probability of calling false SNPs and assigning incorrect genotypic states. Such genotyping criteria are based on relatively high depth requirements, information again accessible for evaluation via the master matrix.

Through its strict initial parsing and filtering of the raw reads as well as its rigorous approach to alignment, SNP filtering, and genotyping, GBS-SNP-CROP takes a very conservative approach to SNP calling. Nevertheless, as shown in the next section, the number of identified SNPs compares favorably to more permissive pipelines, in part because of GBS-SNP-CROP’s ability to make use of all available data, regardless of read length.

Finally, in addition to the embedded strategies for minimizing false SNP calls discussed here, users can easily impose additional desired filters due to the fact that the output from all GBS-SNP-CROP steps, like the master matrix, are human-readable text files. For example, for the purpose of mapping studies as opposed to diversity analyses, which are the primary focus here, the elimination of markers in particularly SNP-dense regions may be an important quality control, as such high SNP density may be an artifact of promiscuous alignment, particularly in polyploids. In a reference-based approach, such culling is straightforward given the set of unique SNP coordinates across the linkage groups. In a reference-independent pipeline, a similar filter can be applied; but users will need to consider SNP densities within each cluster (centroid) used to build the Mock reference. To accomplish this, centroid boundaries must be located within the Mock Reference, which is one reason why the second Mock Reference (clusters) file is generated by the pipeline, to enable such projection.

### GBS-SNP-CROP performance

We assessed the performance of GBS-SNP-CROP in genotyping a population of 48 diverse accessions of the perennial dioecious tetraploid *Actinidia arguta*. Specifically, its performance using both a reference from a related diploid species (*A. chinensis*) and a Mock Reference was compared to that of TASSEL-GBS [30], a widely-used reference-based pipeline, and TASSEL-UNEAK [15], its reference-independent version.

#### Sampling strategy to build a Mock Reference

Three different GBS-SNP-CROP Mock Reference assembly strategies were investigated, differing only in the numbers of genotypes from the target population used to construct the Mock Reference. Contrary to our original expectations, we found that the number of genotypes used to build the Mock Reference is inversely related to the number of mapped reads retained by the pipeline and thus the number of SNPs called (Table 2). For example, using all reads from the full set of 48 unique genotypes, the pipeline called 14,712 SNPs (average depth = 70.7) that passed all population-level filters. Because more than 4 h were needed to assemble the Mock Reference in this case (see Table 1), we investigated the relative performance of the pipeline under scenarios where fewer genotypes were used to construct the Mock Reference, first using only the top five genotypes (ranked simply by the number of parsed reads) and then again using only the top genotype. Using the five genotypes with the highest numbers of parsed reads, the pipeline assembled the Mock Reference in less than an hour and identified 20,226 potential SNPs (average depth = 71.0). Using only the single most read-abundant genotype, the pipeline assembled the Mock Reference in 14 min and called 21,318 SNPs (average depth = 69.3). Based on these results, all subsequent pipeline evaluation was conducted using the results from GBS-SNP-CROP-MR01 (i.e. Mock Reference constructed from one genotype). The pipeline itself is flexible, however, able to integrate centroids from multiple genotypes into a Mock Reference, a feature of potential use for genotyping particularly diverse populations (e.g. multiple closely-related species).

**Table 2:** Performance of GBS-SNP-CROP under three different sampling strategies for building the Mock Reference: Using all 48 individuals in the population (MR48), using only the 5 individuals with the highest number of parsed reads (MR05), and using only the single most read-abundant genotype (MR01)

Pipelines	Total number of centroids used to build the Mock Reference^a^	Total number of paired-end reads used for SNP calling^b^	Number of SNPs called^c^	Avg. depth^d^	Hetero (%)^e^	Homo (%)^f^	Missing data (%)^g^	Time (hrs:mins)^h^
GBS-SNP-CROP-MR48	1,276,734	92,667,123	14,712	70.74	32.47	59.31	8.20	14:30
GBS-SNP-CROP-MR05	500,795	132,920,383	20,226	71.02	34.50	57.18	8.31	12:06
GBS-SNP-CROP-MR01	229,549	154,506,669	21,318	69.34	34.51	56.85	8.29	11:03

^a^ Total number of non-redundant consensus sequences (centroids) identified via clustering to represent the GBS fragment space. This is also the number of FASTA entries in the “MockRef_Clusters.fasta” file

^b^ Number of reads retained by the pipeline after mapping procedures and thus used for SNP calling

^c^ Total number of SNPs called, given all SNP calling filters and genotyping criteria described in the text

^d^ Average read depth for all SNPs across the entire population

^e^ Percentage of heterozygous genotype calls

^f^ Percentage of homozygous genotype calls

^g^ Percentage of missing cells (i.e. no genotype call for a given SNP*accession combination) in the final SNP genotype matrix

^h^ The total computation time required for all pipeline analysis when executed on a Unix workstation with 16 GB RAM and a 2.6 GHz Dual Intel processor

#### Data usage

One of the most noteworthy differences between the GBS-SNP-CROP and TASSEL pipelines is the ability of GBS-SNP-CROP to access and make use of a greater amount of sequence data (Table 3). In the TASSEL-GBS pipeline, due to its tag-based alignment strategy, a uniform tag length (mxTagL) must be specified that effectively limits the number of reads used for analysis. According to the TASSEL 5.2.11 manual, “the mxTagL value must be chosen such that the longest barcode + mxTagL < read length” [30]; thus all reads that violate this statement are discarded. Further, all reads that meet this requirement are subsequently truncated to a uniform length based on this parameter; thus not only short reads but also the full lengths of long reads are culled. While this tag length requirement is adjustable within TASSEL-GBS (here, we ran the pipeline with tag lengths of both 32 bp as well as the default 64 bp), it is fixed at 64 bp for TASSEL-UNEAK. In contrast, aside from a user-specified minimum read length, GBS-SNP-CROP imposes no requirement for read length uniformity, even within read pairs.

**Table 3:** Comparative data usage and computation times for five different analyses of 150 bp paired-end GBS data from 48 accessions of *Actinidia arguta*

Pipeline	Min required read length (bp)^a^	Max usable read length (bp)^b^	Total number of usable R1 reads^c^	Total usable bases (Gb)^d^	Time (hrs:mins)^e^
*Reference-based*					
GBS-SNP-CROP-RG	32	NA	128,577,030	16.82	8:30
TASSEL-GBS-mxTagL32	50	32	120,593,880	3.85	0:35
TASSEL-GBS-mxTagL64	75	64	105,908,174	6.77	1:10
*Reference-independent*					
GBS-SNP-CROP-MR01	32	NA	128,577,030	16.82	11:03
TASSEL-UNEAK	32	64	134,352,640	8.60	0:27

^a^ GBS-SNP-CROP utilizes the entire R1 and R2 paired-end sequences of all parsed and quality trimmed reads longer than a user-specified (i.e. adjustable) minimum length, in this case 32 bp. The TASSEL-GBS pipelines utilize a uniform user-specified portion (e.g. 32 bp, 64 bp) from the beginning of acceptable R1 (single-end) reads that exceed a minimum length (e.g. 50 bp, 75 bp) before barcode and cut site trimming. TASSEL-UNEAK utilizes up to 64 bp from the beginning of acceptable R1 (single-end) reads that exceed a minimum length of 32 bp after barcode and cut site trimming

^b^ The maximum length of sequences utilized by GBS-SNP-CROP is set by the sequencing platform (e.g. 100 bp, 150 bp, etc.). In TASSEL-GBS, the user specifies a maximum tag length, thereby effectively setting a uniform tag length. The maximum usable sequence length in TASSEL-UNEAK is 64 bp, with all shorter reads greater than 32 bp padded with poly-A’s to a uniform 64 bp tag length

^c^ The number of R1 (i.e. single-end) reads ultimately used by each pipeline, after filtering based on quality and read length requirements. The R1 (single-end) counts are shown here to facilitate comparison across pipelines. Because GBS-SNP-CROP utilizes paired-end reads, the total number of actual reads used (R1 and R2) is twice this number

^d^ The total number of nucleotides of sequence data used in each analysis

^e^ The total computation time required for each analysis when executed on a Unix workstation with 16 GB RAM and a 2.6 GHz Dual Intel processor

Following initial parsing and quality trimming (Stage 1), a total of 16.82 Gb of sequence data was found to be usable for analysis (alignment, SNP discovery, etc.) within GBS-SNP-CROP (Table 3). In contrast, due mainly to tag length requirements and the usability of only R1 (single-end) reads, a much reduced 3.85 Gb, 6.77 Gb and 8.60 Gb were used, respectively, by the TASSEL-GBS (mxTagL = 32), TASSEL-GBS (mxTagL = 64) and TASSEL-UNEAK pipelines. In terms of data usage, therefore, GBS-SNP-CROP performs quite favorably, with approximately 2.0–4.4 times more high-quality sequence data available to it for SNP discovery.

In theory, one should be able to make more reads available to TASSEL-GBS by reducing the mxTagL threshold. Such a reduction (in this case, from 64 to 32 bp) leads, however, to a significant reduction in overall data usage (from 6.77 Gb to 3.85 Gb; Table 3) and a concomitant reduction in identified SNPs (from 8,907 to 5,593; Table 4B). For TASSEL-GBS, therefore, it may be advantageous to set a larger mxTagL value, thereby discarding a larger number of reads that fail to meet that requirement, than to use a higher number of shorter reads permitted by a lower mxTagL value.

**Table 4:** Comparative pipeline performances before (4A) and after (4B) depth-based genotyping criteria and population-level SNP calling filters for 150 bp paired-end GBS data from 48 accessions of *Actinidia arguta*

Pipeline	Number of SNPs^a^	Average depth [D]^b^	Reads with D ≥ 20 (%)^c^	Hetero (%)^d^	Homo (%)^e^	Missing data (%)^f^
4A. No SNP calling or genotyping filters applied^g^						
GBS-SNP-CROP-MR01	56,598	44.47	52.85	26.19	57.25	16.55
GBS-SNP-CROP-RG	23,564	47.39	39.00	26.10	51.09	22.80
TASSEL-UNEAK	12,905	6.98	9.61	13.93	52.12	33.94
TASSEL-GBS-mxTagL32	19,095	134.18	49.20	16.86	71.66	11.46
TASSEL-GBS-mxTagL64	25,005	34.65	35.60	19.21	65.80	14.98
4B. Depth-based genotyping criteria and population-level SNP calling filters applied^h^						
GBS-SNP-CROP-MR01	21,318	69.34	99.92	34.51	56.85	8.64
GBS-SNP-CROP-RG	5,471	77.11	99.85	38.31	53.29	8.40
TASSEL-UNEAK	1,160	44.70	83.62	31.66	66.61	1.73
TASSEL-GBS-mxTagL32	5,593	64.41	92.52	26.33	71.58	2.09
TASSEL-GBS-mxTagL64	8,907	51.42	78.07	27.80	69.36	2.84

^a^ Total number of SNPs called within each pipeline, under the indicated SNP calling filters and genotyping criteria

^b^ Average read depth for all SNPs across the entire population

^c^ Percentage of called SNPs with an average read depth of at least 20

^d^ Percentage of heterozygous genotype calls

^e^ Percentage of homozygous genotype calls

^f^ Percentage of missing cells (i.e. no genotype call for a given SNP*accession combination)

^g^ Liberal pipeline results in the absence of subsequent SNP calling or genotyping filters

^h^ Pipeline results after culling SNPs with less than 75 % scored genotypes, with D ≤ 4 (low depth), or D ≥ 200 (over-represented sequences). Further reduction is due to imposing stringent depth-based genotyping criteria, including a minimum read depth of 11 for homozygotes when the secondary allele count is zero, a minimum depth of 48 for homozygotes when the secondary allele count is one, a minimum depth of 3 for each allele for heterozygotes, and a read-depth ratio of the lower- to higher-depth allele greater than 0.1

**Fig. 3:**
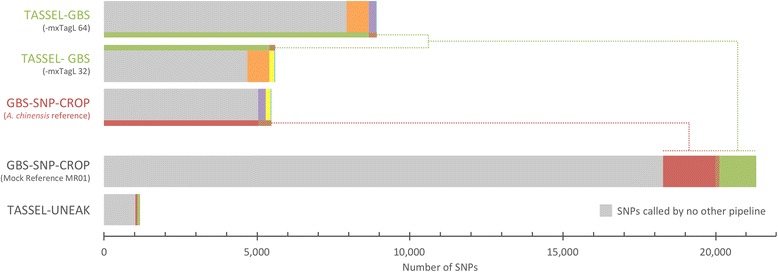
Bar plot showing the extent of marker overlap among the five evaluated pipelines. The sets of SNPs called by the five pipelines are largely orthogonal to one another, as shown by the fact that both the reference-based and reference-independent pipelines call high proportions of SNPs called by no other pipeline (grey bars). Shared SNPs among pipelines are indicated by color-coordinated bars. Whereas only 0.6 and 0.4 % of the 8,907 and 5,593 SNPs called by TASSEL-GBS-64 and TASSEL-32, respectively, were identified by TASSEL-UNEAK, 33.7 % of the SNPs called by GBS-SNP-CROP-RG were called by GBS-SNP-CROP-MR01

The low average proportion (10.8 %) of shared SNPs discovered by TASSEL-GBS under both the 32 and 64 bp mxTagL scenarios (Fig. 3; Additional file 2) indicates that essentially different datasets are made available to the TASSEL-GBS pipeline, depending on the chosen value of this one parameter. Such a comparison suggests that the requirement within the TASSEL pipelines for uniform read lengths (i.e. TASSEL’s tag-based mapping strategy) is fundamentally limiting, in terms of data usage. By taking a read-based rather than a tag-based approach to alignment and SNP discovery, GBS-SNP-CROP leverages all available data in a single analysis, thereby avoiding undue fractionation of the dataset.

#### Numbers of SNPs

Analyses by the different pipelines lead to widely varying numbers of identified SNPs (Table 4). Using only the single most read-abundant genotype to build the Mock Reference, GBS-SNP-CROP called 56,598 potential SNPs (average depth = 44.5; Table 4A), of which 21,318 were retained after applying all SNP calling and genotyping filters (Table 4B), a reduction of 62.3 %. In comparison, the reference-free TASSEL-UNEAK pipeline called 12,905 potential SNPs (average read depth = 7.0), of which only 1,160 SNPs passed these same filters, a striking reduction of 91.0 %.

Using the published *A. chinensis* diploid genome as a reference and a liberal pipeline (i.e. no imposed SNP culling or genotyping filters), GBS-SNP-CROP-RG called 23,564 potential SNPs (average depth = 47.4), of which 5,471 were retained after filtering, a reduction of 76.8 %. In comparison, the 32 and 64 bp reference-based TASSEL-GBS analyses called 19,095 and 25,005 potential SNPs (average depths of 134.2 and 34.7, respectively), of which 5,593 (70.7 % reduction) and 8,907 (64.4 % reduction) passed the imposed filters (Table 4). Unlike the reference-independent analyses, therefore, TASSEL-GBS was found to outperform the reference-based GBS-SNP-CROP in terms of numbers of identified SNPs.

Using only those SNPs that passed the stringent genotyping criteria and population-level filters described earlier, we compared the set of SNPs called by GBS-SNP-CROP (using the *A. chinensis* reference) with those called by the TASSEL-GBS analyses (Table 4B). There is strikingly little congruence among these analyses, with many unshared markers (on average 96.3 %) between them (Fig. 3; Additional file 2). Interestingly, a high proportion of unshared markers (on average 89.2 %) also exists between the two different TASSEL-GBS analyses themselves, even though they differ only in their specified mxTagL thresholds. Because the initial dataset is the same for both TASSEL analyses, we expected roughly half of the SNPs called under mxTagL = 64 to also be called under mxTagL = 32 (i.e. that SNPs located within the first 32 bases of the mxTagL = 64 SNPs should comprise a proportional subset of the mxTagL = 32 SNPs); but such is not the case (see Fig. 3).

One stated reason for TASSEL’s approach to SNP calling based on tags is decreased computational time spent for pipeline execution, with the added rationale that sequencing errors increase after the first 64 bp of a read [11, 30]. While this may be the case, TASSEL’s SNP discovery method appears to be highly sensitive to this tag length parameter, a result that suggests there may be some benefit in aggregating the results (i.e. lists of SNPs) of multiple TASSEL-GBS analyses under various mxTagL values. Similarly, the largely non-overlapping results of the reference-based GBS-SNP-CROP analysis may also have value as a complement to the TASSEL-GBS approach.

To investigate the overlap among the sets of SNPs called between the reference-based and reference-independent pipelines, we mapped all SNPs discovered using both GBS-SNP-CROP (Mock Reference centroids) and TASSEL-UNEAK (tags) to the *A. chinensis* reference. In so doing, we found that 33.7 % of the SNPs called by the reference-based GBS-SNP-CROP (*A. chinensis*) were also called by the reference-independent GBS-SNP-CROP (Mock Reference based on the single most read-abundant genotype). In contrast, only 0.6 and 0.4 % of the SNPs called by TASSEL-GBS (64 and 32 bp, respectively) were identified by the reference-independent TASSEL-UNEAK pipeline (Fig. 3; Additional file 2).

#### Average depth

One of the most efficient means of distinguishing sequencing error from true nucleotide polymorphism is to increase read depth thresholds because polymorphisms called on the basis of more reads mapped to the same locus can be declared with greater reliability that those based on fewer reads [46]. Nielsen et al. [47] discussed many studies using NGS data with medium-to-low coverage (<20×) and showed that genotype calls based on such data exhibit statistical uncertainty. According to the authors, there are two reasons for this: (1) In heterozygotes, both alleles may not be sampled, thus leading to incorrect homozygote calls; and (2) In the case of high sequencing error technologies, a significant number of homozygotes may be incorrectly declared heterozygotes if genotype calling is based simply on the allelic presence/absence. According to Illumina’s technical notes [35], the probability of making a correct genotyping call is roughly 95 % for 20× coverage. While 99.9 % of the 21,318 SNPs identified by the GBS-SNP-CROP Mock Reference pipeline have an average read depth higher than 20×, this is true of only 83.6 % of the 1,160 SNPs called by TASSEL-UNEAK (Table 4). In comparison, 92.5 % of the 5,593 SNPs (-mxTagL32) and 78.1 % of the 8,907 SNPs (-mxTagL64) called by the reference-based TASSEL-GBS pipelines have an average read depth higher than 20×, compared to 99.9 % of the 5,471 SNPs called by the reference-based GBS-SNP-CROP. In terms of average read-depth, therefore, GBS-SNP-CROP performs favorably compared to both TASSEL-GBS and TASSEL-UNEAK (see Additional file 3).

#### Recognizing biological replicates

The primary motivation for developing GBS-SNP-CROP was the need for a tool to accurately characterize the genetic diversity of understudied germplasm collections, including identifying redundant accessions as a means of boosting the resource efficiency of curation efforts. Given this goal, a relevant performance criterion is the ability of the pipeline to identify biological replicates in a population, as indicated by the observed genetic distance between those replicates. To quantify such distance, we employed a modified Gower’s Coefficient of Similarity [48], ranging from 0 to 1, to quantify identity-by-state based on bi-allelic SNPs:

$$ {S}_{Gower}\left(x,y\right) = \frac{{\displaystyle {\sum}_{i=1}^m}{s}_i{w}_i}{{\displaystyle {\sum}_{i=1}^m}{w}_i} $$

where s_i_ = 1 if the genotypes are the same, 0.5 if the genotypes differ by one allele (i.e. heterozygote vs. homozygote), and 0 if the genotypes differ by both alleles (i.e. primary homozygote vs. secondary homozygote); and w_i_ = 1 if both replicates are genotyped for the SNP in question and 0 if either replicate lacks an assigned genotypic state.

Using the SNPs called by the GBS-SNP-CROP-MR01 analysis (Table 4B), the Gower genetic similarity calculated between two biological replicates of *A. arguta* accession ‘Opitz Male’ was found to be 0.999, with a Pearson correlation of 0.998, results similar to those obtained with the reference-based GBS-SNP-CROP pipeline (Table 5). In comparison, the reference-based TASSEL-GBS-32 bp and -64 bp analyses yielded lower Gower genetic similarities of 0.967, as well as reduced Pearson correlations (≤ 0.92). These same replicates of ’Opitz Male’ were found to be only 0.948 similar by TASSEL-UNEAK, indicating a genotyping error rate of more than 60 times that of GBS-SNP-CROP (Mock Reference), despite calling 18 times fewer SNPs (1,160 vs. 21,318; Table 4B). This same basic pattern of results was found when analyzing biological replicates of *A. arguta* accession ‘Dumbarton Oaks’ (Table 5), suggesting that genotyping via GBS-SNP-CROP is relatively robust, prone to fewer genotyping errors while maintaining high numbers of SNPs, whether or not a reference is available.

**Table 5:** Comparative pipeline performances, in terms of consistency in genotyping biological replicates

		cv. ‘Opitz Male’			cv. ‘Dumbarton Oaks’		
Pipelines	Number of SNPs^a^	Gower genetic similarity^b^	Pearson correlation^c^	Shared genotype calls (%)^d^	Gower genetic similarity	Pearson correlation	Shared genotype calls (%)
GBS-SNP-CROP-MR01	21,318	0.999	0.998	99.9	0.998	0.997	99.8
GBS-SNP-CROP-RG	5,471	0.999	0.998	99.9	0.998	0.997	99.9
TASSEL-UNEAK	1,160	0.935	0.948	93.6	0.950	0.961	94.8
TASSEL-GBS-32 bp	5,593	0.967	0.909	96.3	0.969	0.922	96.4
TASSEL-GBS-64 bp	8,907	0.967	0.920	96.7	0.966	0.919	96.6

^a^ The total number of SNPs used in this analysis refers to numbers from Table 4B

^b^ A modified Gower’s general Coefficient of Similarity [48], ranging from 0 to 1, to quantify identity-by-state based on bi-allelic SNPs

^c^ Pearson correlation calculated using R [31]; for all correlations in the table, *p-value* < 0.01

^d^ The percentage of SNPs with exact genotype matches for the two biological replicates. All loci with missing data for either replicate were discarded

#### Computation time

Compared to TASSEL-UNEAK, the GBS-SNP-CROP Mock Reference workflow processed over twice as much data, generated over 18 times more SNPs, the SNPs it called had higher average depth (69.3 vs. 44.7), and as a set they were better able to detect similarity between biological replicates; but this improved performance comes at the price of approximately 25 times longer computation time. Using a dedicated Unix workstation with a 2.6 GHz Dual Intel processor and 16 GB RAM, the computational time required to run the Mock Reference GBS-SNP-CROP pipeline using only the most read-abundant genotype to assemble the Mock Reference was approximately 11 h for this dataset, compared to only 27 min for the TASSEL-UNEAK analysis (Table 2). Similarly, due to its consideration of 3–4 times the amount of sequence data and its strategy of mapping reads rather than tags, the reference-based GBS-SNP-CROP analysis (~8.5 h) also requires significantly more computational time than either of the TASSEL-GBS analyses (35–70 min). Table 1 presents the computational times required for each of the steps within the reference-free GBS-SNP-CROP-MR01 workflow.

## Conclusions

GBS-SNP-CROP is a complete bioinformatics pipeline developed to support curation, research, and breeding programs wishing to utilize GBS for the cost-effective genome-wide characterization of plant genetic resources in the absence of a reference genome. Although the pipeline was created primarily with orphan crop characterization in mind, its underlying strategy is sufficiently general to suggest its potential utility in any situation (plant, animal, or micro-organismal) where reduced-representation genomic data (e.g. GBS) is analyzed for SNPs, such as studies in population genetics, evolutionary ecology, conservation biology, and genetic linkage analysis.

As indicated by the example analysis presented here, the pipeline performs quite favorably compared to TASSEL-UNEAK, not only in terms of a significantly higher number of identified SNPs but also in terms of an increased average read depth and a greatly reduced genotyping error rate. Remarkably, the reference-independent version of GBS-SNP-CROP was also shown to outperform the reference-based TASSEL-GBS pipeline in terms of these same metrics. In contrast, the reference-based version of GBS-SNP-CROP appears outperformed by TASSEL-GBS in terms of the number of called SNPs, though again its genotyping error rate is lower. Given the low proportion of shared SNPs among these reference-based analyses, however, GBS-SNP-CROP may be useful even in this case, able to detect large numbers of additional high-quality SNPs missed by the tag-based and read length-restricted approach of TASSEL-GBS. Indeed, with the capacity to make full use of variable length, paired-end GBS data for high-density SNP genotyping of plant populations, whether or not a reference genome is available, GBS-SNP-CROP is a flexible and easily modifiable tool worthy of consideration by interested programs.

## Methods

### Plant material, GBS data, and genotypes sampled

A collection of 48 tetraploid kiwiberry (*Actinidia arguta*) genotypes, each carrying two sets of 29 chromosomes (2n = 4× = 116) with an estimated total genome size of 1C = 1.5 Gbp [49], was sampled from the USDA National Clonal Germplasm Repository (Davis, CA) for this study. Genomic DNA was extracted from ~1 g of fresh young leaves from each accession using a modified CTAB protocol, and a multiplexed GBS library was prepared according to the two enzyme (*PstI*-*MspI*) protocol described by Poland et al. [11]. Using the first 96 6–10 bp barcodes from that protocol, the 48 accessions were multiplexed along with 2 biological replicates (accessions “Opitz Male” and “Dumbarton Oaks”) and 46 breeding lines, resulting in a 96-plex library which was sequenced on two lanes (i.e. one complete flowcell) of an Illumina 2500 HiSeq machine at the Hubbard Center for Genome Studies, University of New Hampshire (http://hcgs.unh.edu/). FASTQ files of the sequence data were generated using CASAVA 1.8.3 [50]; and these raw sequences have been deposited in the NCBI Sequence Read Archive (SRA Accession number SRR2296676). A table of the 48 genotypes used in this analysis, along with their assigned barcodes, can be found in Additional file 4.

### Pipeline evaluation and testing

To evaluate the performance of GBS-SNP-CROP, we analyzed the GBS data from the 48 accessions described above (plus 2 biological replicates) using seven different analyses. First, we executed three variations of GBS-SNP-CROP without a reference genome (Table 1, Stage 2). In the first Mock Reference analysis (GBS-SNP-CROP-MR48), we assembled the Mock Reference from centroids identified by clustering first within each genotype and then across all 48 genotypes in the population. In the second Mock Reference analysis (GBS-SNP-CROP-MR05), clustering was done across only the five most read-abundant genotypes (accessions “ORUS 2–16”, “DACT 213”, “40537C”, “ORUS 1–6”, and “Chang Bai Mountain 3”). In the third analysis (GBS-SNP-CROP-MR01), the Mock Reference was built using the within-genotype centroids from only the single most read-abundant line (accession “ORUS 2–16”). These three different approaches were followed to examine the effects of reducing the number of genotypes used to build the Mock Reference on both computational time and the number and quality of identified SNPs. For all Mock Reference analyses, we used PEAR v.0.96 [32] to merge reads using mainly default parameters, except for specifying a minimum assembled read length of 32 bp. For clustering, we used USEARCH v.8.0.162 [33], specifying the “cluster_fast” algorithm with a nucleotide similarity threshold of 93 % to allow up to two mis-matches within the shortest assembled reads (32 bp).

For comparison with the Mock Reference analyses described above, we ran GBS-SNP-CROP using a published reference genome from the closely related diploid (2n = 2× = 58) species *A. chinensis* [51] with an estimated genome size 1C = 758 Mbp [48]. The only difference between this reference-based analysis (GBS-SNP-CROP-RG) and the Mock Reference analyses above is that in the former we skipped Stage 2 (“Build the Mock Reference ”) of the GBS-SNP-CROP workflow (see Table 1).

For both GBS-SNP-CROP analyses, the CASAVA-processed sequence data were subjected to basic quality filtering. Specifically, reads were trimmed based on a sequence of three contiguous bases with an average Phred score Q ≤30, and trimmed reads shorter than 32 bp were culled. These procedures were performed using the Trimmomatic software v.0.33 [31] with the following parameters: LEADING:30 SLIDINGWINDOW:4:30 TRAILING:30 MINLEN:32. Also for both analyses, alignment was carried out using BWA v.0.7.12 [34]; and the resultant alignment files were processed with SAMtools v.1.2 [35].

For the next analysis, we used the Network-Based SNP Discovery Protocol with no reference genome (TASSEL-UNEAK v.3.0). The TASSEL-UNEAK pipeline was run using mainly its default parameters, with two changes: (1) In the “UMergeTaxaTagCountPlugin” step, the “-c” flag was increased from 5 to 10; and (2) The error tolerance rate (“-e” flag on “UTagCountToTagPairPlugin”) was decreased from 0.03 to 0.01. These modifications were made in an effort to match the default parameters of the TASSEL-GBS analyses, thereby facilitating comparison.

Finally, we used TASSEL-GBS v.5.2.11 to carry out two more reference-based analyses, one with “Maximum Tag Length” (mxTagL) = 32 bp and the other with mxTagL = 64 bp. For all TASSEL analyses (TASSEL-GBS-32 bp, TASSEL-GBS-64 bp, and TASSEL-UNEAK), we set the minimum minor allele frequency to 5 % and accepted only those markers for which genotypes were called for at least 75 % of the population.

### Comparing called SNPs among pipelines

Identifying shared and non-shared SNPs called by the reference-based pipelines (GBS-SNP-CROP-RG and the TASSEL-GBS pipelines) is straightforward due to the unique coordinate positions of the SNPs within the common *A. chinensis* reference genome. Comparing called SNPs between the reference-independent pipelines (TASSEL-UNEAK and GBS-SNP-CROP-MR01) and reference-based pipelines is less simple due to the fact that no common reference (and thus coordinate system) exists. To enable such important comparisons, we first located the positions of all called SNPs (Table 4B) within the individual centroids used to construct the Mock Reference (GBS-SNP-CROP-MR01) and within the unique 64 bp tags used within the TASSEL-UNEAK pipeline. We then mapped all the putative SNP-containing centroids/tags to the *A. chinensis* reference genome and located the corresponding *A. chinensis* coordinate position of each called SNP. Finally, the allele compositions of any supposedly common SNPs were verified before such SNPs were declared as shared between pipelines.

### Availability of supporting data

The data set supporting the results of this article is available in the NCBI Sequence Read Archive [SRA Accession number SRR2296676].

## Additional files

Additional file 1:
**Suggested parameter values for GBS-SNP-CROP, based on ploidy scenarios and confidence considerations.** *AdditionalFile1.pdf* presents rationale to guide user selection of ploidy-appropriate values for various parameters in Script 7 of GBS-SNP-CROP (-mnHoDepth0, -mnHoDepth1, -mnAlleleRatio, and –altStrength). (PDF 1231 kb) Additional file 2:
**Bubble plot showing the pair-wise percentage of shared markers called by the different pipelines.** *AdditionalFile2.pdf* complements the visualization in Fig. 3, showing all pair-wise proportions of shared SNPs among the five evaluated pipelines. (PDF 732 kb) Additional file 3:
**The distribution of pre-filtered SNPs across three different depth classes: Low (<4), Acceptable (4–200), and Over-represented (>200).** The bar plot in *AdditionalFile3.pdf* compares the distributions of average read depths for the pre-filtered SNPs called by the five evaluated pipelines. (PDF 43 kb) Additional file 4:
**List of the 48** ***A. arguta*** **genotypes used for GBS-SNP-CROP development and analysis.** *AdditionalFile4.pdf* presents the names of the genotypes from the USDA National Clonal Germplasm Repository used in this study, along with their barcodes and the number of parsed GBS reads obtained for each. (PDF 50 kb)
