# Supplementary material for: GBS-SNP-CROP: a reference-optional pipeline for SNP discovery and plant germplasm characterization using variable length, paired-end genotyping-by-sequencing data
Source: BMC Bioinformatics. 2016 Jan 12;17:29. doi: 10.1186/s12859-016-0879-y (PMC4709900; doi:10.1186/s12859-016-0879-y)
Supplement: Additional file 1: — Suggested parameter values for GBS-SNP-CROP, based on ploidy scenarios and confidence considerations. AdditionalFile1.pdf presents rationale to guide user selection of ploidy-appropriate values for various parameters in Script 7 of GBS-SNP-CROP (-mnHoDepth0, -mnHoDepth1, -mnAlleleRatio, and –altStrength). (PDF 1231 kb) [file 12859_2016_879_MOESM1_ESM.pdf]

Additional file 1: Suggested parameter values for GBS-SNP-CROP, based on ploidy scenarios and confidence considerations.

Various user-defined parameters within Script 7 of the GBS-SNP-CROP workflow should be adjusted depending on the ploidy of the species under study (i.e. the number of independent copies of a SNP locus in the genome) and the desired level of error control of the user. The following rationale and the summary table at the end of this Additional file are intended to guide users in their selection of these values:

–mnHoDepth0 and –mnHoDepth1

Assuming random (i.e. non-biased) allele sampling during GBS library preparation and sequencing, the maximum probability for any given heterozygous locus that a sequenced GBS fragment will capture the primary allele is:

$$P(sequencing\ the\ primary\ allele) = \frac{p-1}{p}$$

where p is the ploidy of the species (i.e. p = 2 for a diploid, 4 for a tetraploid, 6 for a hexaploid, etc.). The probability that the primary allele will be sampled d (for *d*epth) independent times is therefore:

$$P(sequencing\ the\ primary\ allele\ d\ times) = \left(\frac{p-1}{p}\right)^d$$

Thus the probability that the alternate allele will be sampled at least *once* over d independent samples is:

$$P(sequencing\ the\ alternate\ allele\ at\ least\ once\ in\ d\ samples) = 1 - \left(\frac{p-1}{p}\right)^d$$

For any single genotype call (i.e. for any single SNP-accession combination), it reasonable that a user would want this probability of detecting the alternate allele, if it exists, to be at least 95%. For the tetraploid case (p = 4), if one wishes to call a homozygote with at 95% confidence, this requirement dictates a minimum read depth of at least:

$$d_{min} = \frac{\ln{(1-0.95)}}{\ln{(\frac{p-1}{p})}} = \frac{\ln{(1-0.95)}}{\ln{(0.75)}} = 10.4 \rightarrow 11$$

Calculated in this way, this minimum depth required for calling homozygotes should be considered an absolute minimum because it controls error only in the case of a single genotypic call, whereas actual GBS datasets may require $>10^6$ such calls, if not more. To control error across the entire set of such calls (i.e. to permit, say, only 1 erroneous homozygote declaration in 10^6 calls), the minimum required depth increases significantly:

$$d_{min} = \frac{\ln{(10^{-6})}}{\ln{(0.75)}} = 48$$

Which values are chosen for homozygote depth thresholds depends both on ploidy as well as on the user's attitude toward error control. For this manuscript (48 accessions of 4x kiwiberry), we set *–mnHoDepth* = 11 to call homozygotes in the absence of any reads of the alternative (i.e. secondary) allele but used the higher threshold (*-mnHoDepth1* = 48), commensurate with the size of our dataset, when calling a homozygote in the presence of a single read of the alternate allele, such higher error control being needed to dismiss this single read as an error.

-mnAlleleRatio

At low read depth (e.g. the d_{min} = 11 case above), the minimum acceptable ratio of the depth of the secondary (2°) allele to that of the primary (1°) allele (*-mnAlleleRatio*) can be inferred directly, namely:

$$2°:1°_{min} = \frac{1}{d_{min} - 1} = \frac{1}{10} = 0.1$$

Simulations indicate that this cutoff ratio increases in value as overall read depth increases (e.g. for a tetraploid, this value increases to 0.25 [95% confidence] for a read depth of 200); therefore, the calculated value above should once again be considered an absolute minimum for users of this pipeline.

-altStrength

Another parameter with clear ploidy dependency is *–altStrength*, the minimum required proportion of secondary reads to all non-primary reads [i.e. 2° allele depth / (2° + 3° + 4° depths)]. In the tetraploid case, assuming an acceptable minimum 0.1 allele depth ratio as calculated above and an upper boundary on sequencing error of 10 errors per kbp [39,40], the suggested minimum value of *–altStrength* = 0.1 / (0.1 + 0.01) = 0.9.

With the above rationale and description as a guide, users will hopefully find the following table useful as a rough guide in setting appropriate values for GBS-SNP-CROP Script 7 parameters, based on their study and objectives:

	Error rate	0.05	0.01	0.001	0.0001	0.00001	0.000001	*-mnAlleleRatio (min values)*	*-altStrength (min values)*
	Confidence	95%	99%	99.9%	99.99%	99.999%	99.9999%		
ploidy (p)	2	5	7	10	14	17	20	0.25	0.962
	4	11	17	25	33	41	48	0.10	0.909
	6	17	26	38	51	64	76	0.063	0.862
	8	23	35	52	69	87	104	0.045	0.820

↑ *-mnHoDepth0 (min values)* (95% column)

↑ *-mnHoDepth1 (for datasets on order of 10^6 calls)* (99.9999% column)
