# Supplementary material for: GBS-SNP-CROP: a reference-optional pipeline for SNP discovery and plant germplasm characterization using variable length, paired-end genotyping-by-sequencing data
Source: BMC Bioinformatics. 2016 Jan 12;17:29. doi: 10.1186/s12859-016-0879-y (PMC4709900; doi:10.1186/s12859-016-0879-y)
Supplement: Additional file 2: — Bubble plot showing the pair-wise percentage of shared markers called by the different pipelines. AdditionalFile2.pdf complements the visualization in Fig. 3, showing all pair-wise proportions of shared SNPs among the five evaluated pipelines. (PDF 732 kb) [file 12859_2016_879_MOESM2_ESM.pdf]

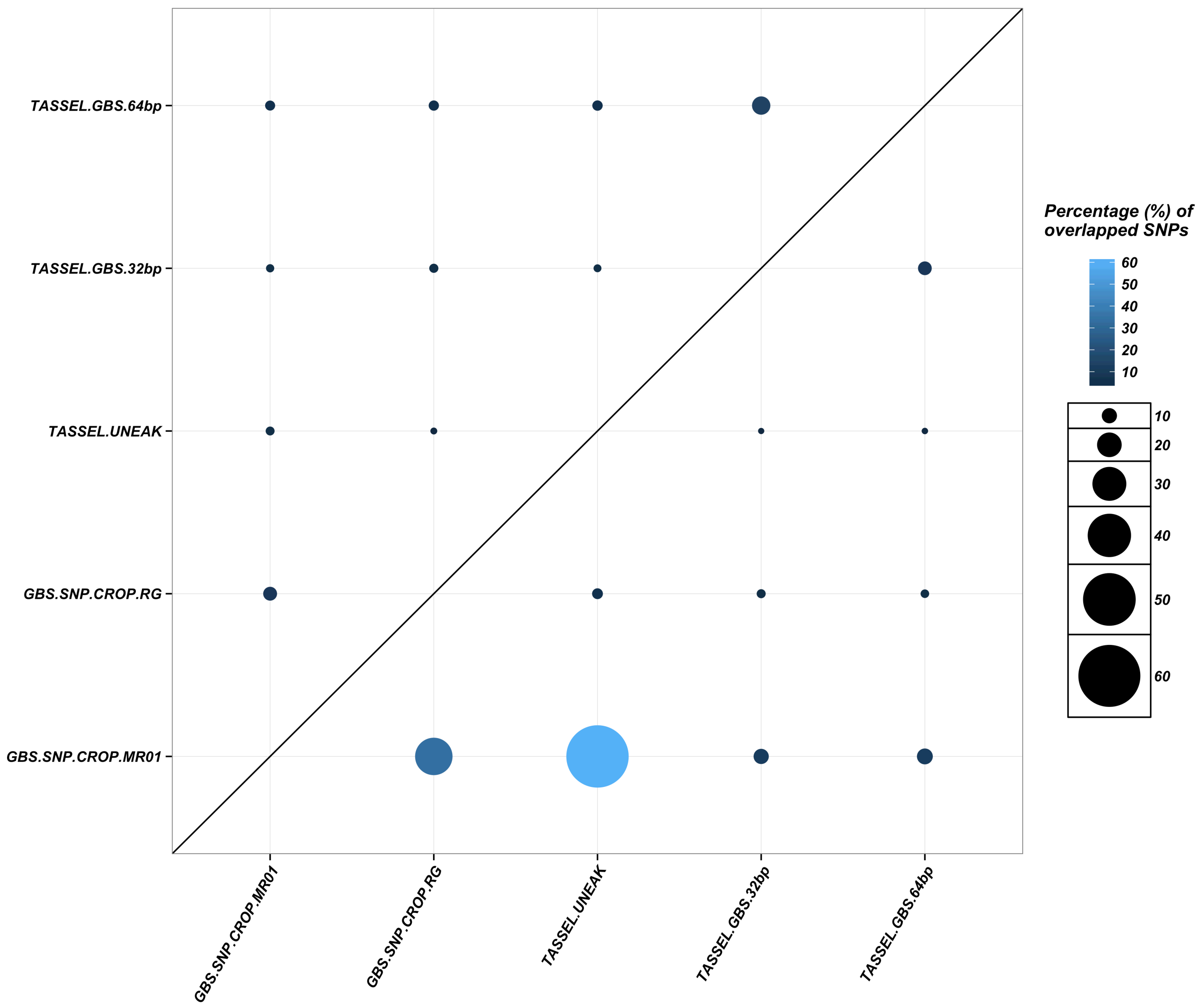

Additional file 2: Bubble plot showing the pair-wise percentage of overlapping markers called by the different pipelines. This plot complements the visualization in Figure 3, showing all pair-wise proportions of shared SNPs among the five evaluated pipelines. This plot indicates, for example, that GBS-SNP-CROP-MR01 calls 33.7% (1,844 SNPs) of the 5,471 SNPs called by GBS-SNP-CROP-RG; but those same 1,844 SNPs comprise only 8.6% of the 21,318 SNPs called by GBS-SNP-CROP-MR01.
