# Supplementary material for: GBS-SNP-CROP: a reference-optional pipeline for SNP discovery and plant germplasm characterization using variable length, paired-end genotyping-by-sequencing data
Source: BMC Bioinformatics. 2016 Jan 12;17:29. doi: 10.1186/s12859-016-0879-y (PMC4709900; doi:10.1186/s12859-016-0879-y)
Supplement: Additional file 3: — The distribution of pre-filtered SNPs across three different depth classes: Low (<4), Acceptable (4–200), and Over-represented (>200). The bar plot in AdditionalFile3.pdf compares the distributions of average read depths for the pre-filtered SNPs called by the five evaluated pipelines. (PDF 43 kb) [file 12859_2016_879_MOESM3_ESM.pdf]

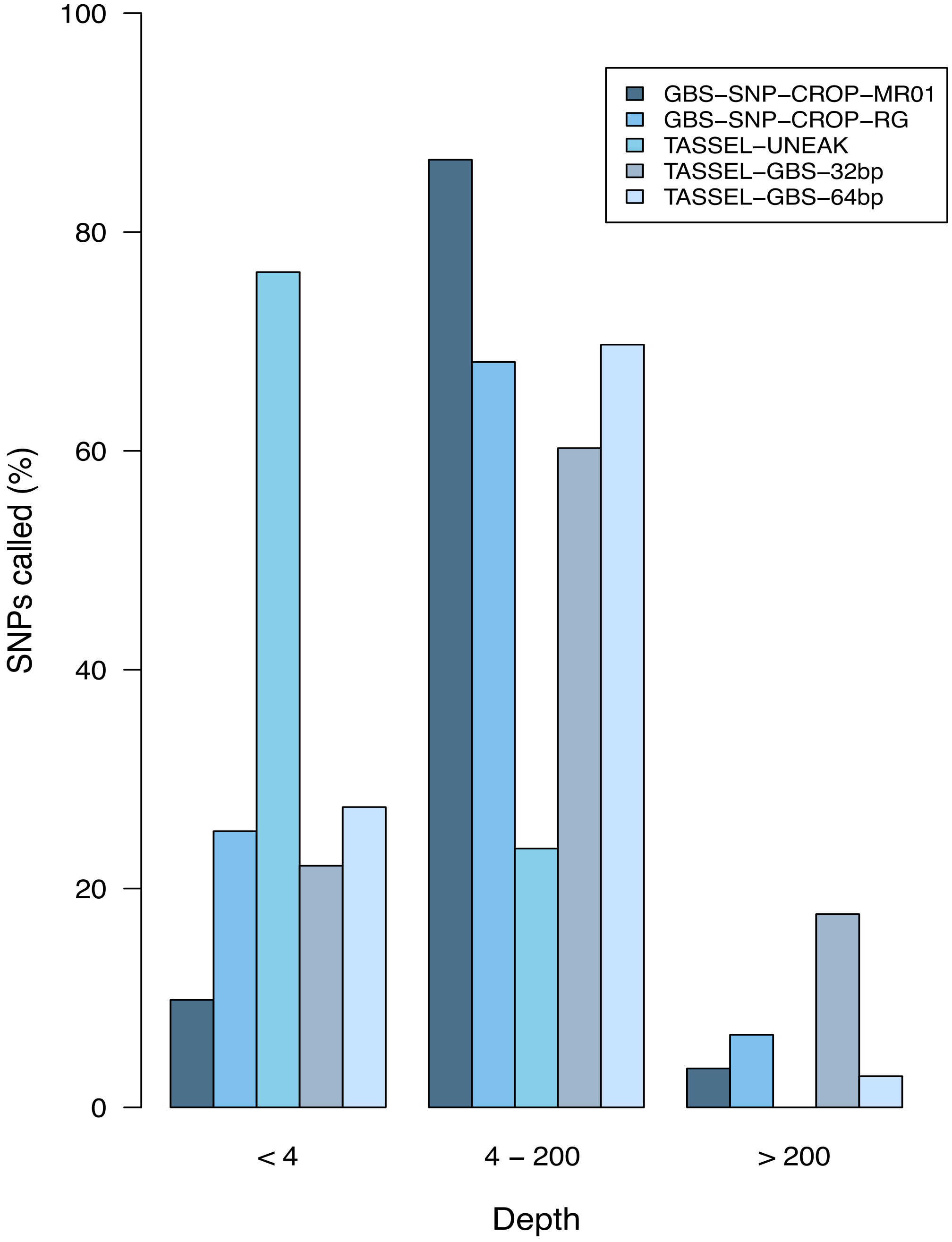

Additional file 3: The distribution of pre-filtered SNPs across three different depth classes: Low (<4), Acceptable (4-200), and Over-represented (>200). The plot compares the distributions of average read depths for the pre-filtered SNPs called by the five evaluated pipelines (Table 4A).
