# Supplementary material for: GBS-SNP-CROP: a reference-optional pipeline for SNP discovery and plant germplasm characterization using variable length, paired-end genotyping-by-sequencing data
Source: BMC Bioinformatics. 2016 Jan 12;17:29. doi: 10.1186/s12859-016-0879-y (PMC4709900; doi:10.1186/s12859-016-0879-y)
Supplement: Additional file 4: — List of the 48 A. arguta genotypes used for GBS-SNP-CROP development and analysis. AdditionalFile4.pdf presents the names of the genotypes from the USDA National Clonal Germplasm Repository used in this study, along with their barcodes and the number of parsed GBS reads obtained for each. (PDF 50 kb) [file 12859_2016_879_MOESM4_ESM.pdf]

Additional file 4: List of the 48 *A. arguta* genotypes used for GBS-SNP-CROP development and analysis. The genotypes from the USDA National Clonal Germplasm Repository used for this study, along with their barcodes (SRA Accession number SRR2296676) and the number of parsed GBS reads obtained for each from Stage 1 of the GBS- SNP-CROP workflow.

**Number**	**Genotype ID**	**Barcode sequence**	**Number of parsed Paired-End reads**
1	Cherry Bomb	TGACGCCA	7413440
2	[5] Chang Bai Mountain 3	CAGATA	11533362
3	[2] DACT 213	GAAGTG	11937552
4	[1] ORUS 2-16	TAGCGGAT	12638534
5	[3] 40537-C	TATTCGCAT	11914118
6	Meader Male	CCGAACA	5928514
7	ORUS 1-3	GGAAGACAT	3857518
8	Ananasnaya	AACGCACATT	3889648
9	Chang Bai Mountain 4	CCTTGCCATT	8735374
10	[BR] Opitz Male	GGTATA	5665492
11	[BR] Opitz Male	TCTTGG	6587636
12	Frenchman's Bay	GGTGT	6368934
13	ORUS 2-17	GGATA	2219262
14	Chang Bai Mountain 5	ACTGCGAT	1855156
15	Michigan State	TTCGTT	2299744
16	74-46	TGGCAACAGA	1719752
17	Early Cordifolia	CTCGTCG	4386424
18	Cornell	GCCTACCT	3747072
19	[BR] Dumbarton Oaks	GGAACGA	4174406
20	74-55	ACTGCT	3007282
21	Jumbo	CGTGGACAGT	3264126
22	ORUS 1-4	TGCTT	4645838
23	74-9	CGCACCAATT	2766536
24	Chang Bai Mountain 2	CTCGCGG	3433118
25	Cordifolia	AACTGG	2630926
26	HVSC-115	ATGAGCAA	2840748
27	DACT 216	CTTGA	5630854
28	New Zealand	GCGTCCT	5886234
29	Geneva 3	ACCAGGA	3004690
30	Langer	CCACTCA	5397382
31	[BR] Dumbarton Oaks	TCACGGAAG	3955312
32	Issai small fruit variant	ATATCGCCA	3151572
33	Meader (Paul Klassen)	CTCTA	5185634
34	74-32	GGTGCACATT	5488740
35	West	CAGAGGT	1264734
36	Chico	AATGAACGA	3305514
37	Issai	GAAGCA	4525900
38	Hardy Red	AACGTGCCT	4646988
39	Geneva 2	CCTCG	2920130
40	#211 Male	GCGCCG	6017050
41	DACT 217	TAGATGA	5626158
42	ORUS 2-1	TTGCTG	2556928
43	ORUS 1-8	ACAGT	2096570
44	[4] ORUS 1-6	CATAT	11782074
45	ORUS 2-7	CATCTGCCG	2135604
46	ORUS 3-3	GGACAG	8156828
47	Turrets Male	AAGACGCT	2367032
48	Geneva 1	ATCCG	2817216
49	Meader	CTTAG	3917364
50	HVSC-117	CAACCACACA	3209702

[BR] Biological Replicate; [1-5] Ranks, in terms of total number of parsed PE reads
